# Comparison of Motor Vehicle Collision Injuries between Pregnant and Non-Pregnant Women: A Nationwide Collision Data-Based Study

**DOI:** 10.3390/healthcare9111414

**Published:** 2021-10-21

**Authors:** Soonho Koh, Masahito Hitosugi, Shingo Moriguchi, Mineko Baba, Seiji Tsujimura, Arisa Takeda, Marin Takaso, Mami Nakamura

**Affiliations:** 1 Department of Legal Medicine, Shiga University of Medical Science, Otsu 520-2192, Japan; millerttime60@gmail.com (S.K.); opi717@belle.shiga-med.ac.jp (S.M.); arisa204@belle.shiga-med.ac.jp (A.T.); marint@belle.shiga-med.ac.jp (M.T.); mamin@belle.shiga-med.ac.jp (M.N.); 2 Center for Integrated Medical Research, Keio University School of Medicine, 35 Shinanomachi, Tokyo 160-8582, Japan; mineko@keio.jp; 3 Joyson Safety Systems Japan K.K. Echigawa Plant, 658 Echigawa, Aisho-cho 529-1388, Japan; Seiji.Tsujimura@jp.joysonsafety.com

**Keywords:** motor vehicle collision, vehicle passenger, pregnant woman, injury, intervention, collision database

## Abstract

We compared the independent predictive factors for moderate and severe injuries, along with characteristics and outcomes of motor vehicle collisions, between pregnant and non-pregnant women. Using 2001–2015 records from the National Automotive Sampling System/Crashworthiness Data System, we selected 736 pregnant women and 21,874 non-pregnant women having any anatomical injuries. Pregnant women showed less severe collisions, fewer fatalities, and less severe injuries in most body regions than non-pregnant women. In pregnant women, the rate of sustaining abbreviated injury scale (AIS) scores 2+ injuries was higher for the abdomen only. For non-pregnant women, rear seat position, airbag deployment, multiple collisions, rollover, force from the left, and higher collision velocity had a positive influence on the likelihood of AIS 2+ injuries, and seatbelt use and force from the rear had a negative influence. There is a need for further development of passive safety technologies for restraint and active safety features to slow down vehicles and mitigate collisions. The influencing factors identified may be improved by safety education. Therefore, simple and effective interventions by health professionals are required that are tailored to pregnant women.

## 1. Introduction

Trauma among pregnant women is common, affecting 1 in 12 pregnancies. Trauma is the leading non-obstetric cause of death among reproductive-age women [1]. According to a systematic review of traumatic injuries among pregnant women, motor vehicle collision (MVC) was the most common and the most life-threatening type of injury [2]. In the United States, approximately 92,500 women have been injured in an MVC and in Japan, 2.9% of pregnant women are involved in MVCs each year [3,4]. After the mother’s involvement in an MVC, negative fetal outcomes often occur. For patients involved in MVCs, there are several factors concerning the collision characteristics that influence fetal outcomes. We previously identified factors influencing fetal outcome in MVCs where detailed information about the collision was available. We found that the maximum abbreviated injury severity (MAIS) score of pregnant woman involved in an MVC was the only significant independent predictor of a negative fetal outcome [5]. Therefore, for the safety of the fetus, measures aimed at decreasing the severity of maternal injury are needed. In our previous study, we also examined factors that predict moderate and severe injuries among pregnant women involved in MVCs. The results suggested that airbag deployment and total changes in vehicle velocity at impact positively influenced, and seatbelt use negatively influenced, moderate-to-severe injuries in pregnant women. To prevent moderate and severe injuries in pregnant women, proper seatbelt use and decreased vehicle velocity are considered effective interventions [5].

Health professionals must also promote safety for all vehicle users. MVC fatalities have increased; there were approximately 1.35 million worldwide in 2016 [6]. Additionally, it has been predicted that MVCs will become the fifth most common cause of fatalities worldwide [6]. Therefore, measures to promote vehicle safety are required to reduce moderate and severe injuries from MVCs, and this includes effective education provided by health professionals. To prevent moderate and severe MVC injuries, if the interventions for pregnant women are different from those for non-pregnant women, specific approaches for pregnant women are needed. However, until now, there have been no studies comparing effective interventions for preventing moderate and severe MVC injuries between pregnant and non-pregnant women.

The objectives of the present study were as follows. First, we aimed to compare the detailed characteristics and outcomes of MVCs involving pregnant and non-pregnant women. Then, we sought to examine independent predictors of moderate-to-severe injuries for non-pregnant women in comparison with those for pregnant women. Finally, we proposed effective measures for both pregnant and non-pregnant women.

## 2. Materials and Methods

### 2.1. Study Design and Patient Selection

This observational study was a retrospective analysis using the data set of the National Automotive Sampling System/Crashworthiness Data System (NASS/CDS). The NASS/CDS is generated by the United States National Highway Traffic Safety Administration. This is a publicity available, de-identified data set that provides data for approximately 5000 collisions every year. The database includes collisions in which at least one of the cars, light trucks, vans, or sports utility vehicles involved was damaged and had to be towed from the scene. The data in each case were collected from interviews with the people involved, police records, medical records, vehicle inspection, scene inspection, and photographs. The raw data can be downloaded via the FTP site of the NASS/CDS [7]. Because of the anonymous and retrospective nature of this study, which used a database open to the public, the requirements for informed consent or approval by an institutional ethics committee were waived.

Collisions involving 141,057 individuals were registered in the NASS/CDS from 2001–2015. We excluded individuals with no anatomical injuries (abbreviated injury scale (AIS) score of 0). We created two data sets for the current study comprising pregnant women and non-pregnant women. For the data on pregnant women, we chose cases involving at least one pregnant occupant. For other cases, the data were further filtered to limit the age of the women to between 15 and 50 years old, which corresponds to childbearing age.

### 2.2. Date Selection

The following information was collected from the database for each person involved in a collision:

(1) General characteristics including age, height, and weight
(2) Seat position (left front, right front, or rear)
(3) Seatbelt use and pretensioner system activation
(4) Airbag deployment
(5) Number of times that the vehicle collided with other vehicles or objects
(6) Principal direction of force. This was according to the clock bearing in degrees for the principal force direction that resulted in the most severe damage in the crash, such as the front of the vehicle at 0 or 360 degrees, the rear of the vehicle at 180 degrees. Then the direction of force was defined as follows: frontal 330 to 30, right lateral 31 to 149, rear 150 to 210, left lateral 221 to 329 degrees;
(7) Total changes in vehicle velocity (delta-V total: DVTOTAL). DVTOTAL was calculated and classified into 10 km/h units by combining the lateral and longitudinal delta-V determined at the inspection.
(8) Rollover
(9) Severity of occupant injury, described using AIS score. The AIS score is used to categorize the injury type and anatomical severity in each body region using a scale from 1 (minor) to 6 (clinically untreatable) [8]. If a woman had multiple injuries in the same region, this corresponded to the maximum score. MAIS was defined as the highest values of AIS score of all body regions for each woman.
(10) Outcome (death, hospital admission, or outpatient).

### 2.3. Statistical Analysis

The data were summarized in the form of values with proportions or frequencies for categorical variables. To summarize continuous variables, we used mean ± standard deviation for values that followed a normal distribution and median with interquartile range for values with a non-normal distribution. Chi-square tests were used to compare prevalence between the two groups. To find the differences in values between the groups, the Student t-test was used for values with a normal distribution, and the Mann-Whitney test was conducted for values with a non-normal distribution. A *p* value < 0.05 was considered statistically significant. To identify which variables were independently associated with substantial injuries in non-pregnant women, we performed multivariable logistic regression analysis. The analyses were performed with IBM SPSS version 23 (IBM Corp., Armonk, NY, USA).

## 3. Results

### 3.1. Comparison between Pregnant and Non-Pregnant Women

During the 15-year study period, data of 736 pregnant women and 21,874 non-pregnant women with an AIS score of 1 or more were collected for analyses. The general characteristics and background of the collisions are summarized in Table 1. Pregnant women were significantly younger, but the difference in age was small. As can be expected, pregnant women had a higher average weight than non-pregnant women. Pregnant women sat in the right front seat (passenger seat) more often and had airbag deployment and pretensioner activation at collision less often than non-pregnant women. Regarding outcome, the number of fatalities was significantly higher in non-pregnant than pregnant women. The prevalence of hospital admission was lower and that of outpatient treatment was higher in non-pregnant women than those in pregnant women (Table 2). Regarding injury severity, the MAIS values were significantly higher in non-pregnant women than in pregnant women. The rates of sustaining AIS 2 or more (AIS 2+) injuries in each body region were compared between the two groups. Only the rate for the abdomen was higher in pregnant women; in other body regions, the rates were higher in non-pregnant women (Table 2). Statistically significant differences were found for the head, chest, spine (abdomen), upper extremities, and lower extremities.

### 3.2. Comparison of Mild and Moderate-to-Severe Injuries in Non-Pregnant Women

We examined the distribution of MAIS in non-pregnant women. Most non-pregnant women (68.9%) had mild injuries with an AIS score of 1, followed by those with MAIS scores of 2 (15.0%), 3 (9.7%), 4 (3.3%), 5 (2.3%), and 6 (0.8%). We divided women into those with mild injuries (MAIS 1) and those with moderate and severe injuries (MAIS 2+). We compared the background and collision characteristics between these two groups (Table 3). Regarding the women’s background, although mean height and weight were significantly higher among women with MAIS 2+ injuries than those with MAIS 1, these differences were negligible (164.6 vs. 164.3 cm, 70.0 vs. 69.3 kg, respectively). Non-pregnant women with AIS 2+ injuries sat in the right front (front passenger) or rear seats significantly more frequently and used a seatbelt significantly less often (*p* < 0.001) than pregnant women. Non-pregnant women with MAIS 2+ had significantly higher vehicle velocities at the time of collision, and more frequent frontal or rear collisions, rollovers, number of collisions, and airbag deployment (*p* < 0.001). Regarding outcomes and injury severity, the prevalence of fatalities and hospital admissions was significantly higher among non-pregnant women with MAIS 2+ injuries than those with MAIS 1 (*p* < 0.001, Table 4).

### 3.3. Factors Influencing Moderate and Severe Injuries in Non-Pregnant Women

To identify variables that were independently associated with moderate and severe injuries among non-pregnant women, we performed multivariable logistic regression analyses with the predictive variables of height, weight, seat position, seatbelt use, airbag deployment, number of collisions, principal direction of force, DVTOTAL, and rollovers. The results showed that height (odds ratio (OR) 1.004), weight (OR 1.005), seat position (rear: OR 1.345), seatbelt use (OR 0.364), airbag deployment (OR 1.297), number of collisions (OR 1.277), principal direction of force (rear: OR 0.360; left: OR 1.478), DVTOTAL (21–40 km/h: OR 2.504; 41–60 km/h: OR 11.674; ≥61 km/h: OR 43.220), and rollover (OR 1.759) were independent predictors of sustaining moderate-to-severe injuries in an MVC (Table 5). According to the Hosmer-Lemeshow test, the suitability of the model was confirmed (*p* = 0.053).

## 4. Discussion

Because several factors concerning collision characteristics influence the mechanisms of injury and outcomes in MVCs, collision details must be considered for vehicle passengers involved in a collision. Large databases have been used to compare characteristics and injury severity between pregnant and non-pregnant women [9,10,11,12]. According to a retrospective cohort study using the Pennsylvania Trauma Outcome Study database, pregnant women had a lower mean injury severity score than non-pregnant women [9]. A retrospective cohort study using the Israel National Trauma Registry suggested that the severity of injuries and the mortality rate among pregnant women involved in MVCs are significantly lower than those of non-pregnant women [10]. A population-based matched retrospective cohort study using the Nationwide Inpatient Database in the United States suggested that pregnant women admitted to hospital following an MVC sustained less severe injuries than non-pregnant women [11]. That study also suggested that the rate of non-pregnant women drivers was higher than that of pregnant drivers [11]. Our results showed that pregnant women had a significantly lower fatality rate and less severe injuries in all body regions, except for the abdomen; these findings were in accordance with the above results.

These past reports lacked detailed information regarding the vehicle collisions, however. The NASS/CDS database includes information such as the collision circumstances and scene, which are useful to determine factors influencing moderate and severe injuries in both pregnant and non-pregnant women. Only one study has previously compared collision characteristics and injury severities between pregnant and non-pregnant women using the NASS-CDS database [12]. In that study, both pregnant and non-pregnant women were most frequently seated on the front left, followed by the front right, and approximately half of them experienced frontal collisions, similar to the present results. However, some results of that study were somewhat different from ours. The study reported that the risk of sustaining MAIS 2+ injuries in pregnant occupants was similar to that for non-pregnant occupants, and crash characteristics were similar between the two groups [12]. Additionally, the study suggested that pregnant women more frequently experienced injuries of AIS 1 or more than non-pregnant women [12]. Pregnant women more frequently used seatbelts than non-pregnant women (65.7% vs. 63.8%), although no significant difference was found in the present study. The rate of airbag deployment was higher in pregnant women than in non-pregnant women (33.8% vs. 23.1%), the opposite pattern to our findings. Differences between the results of that study and the present study could be explained by differences in the sample selection. Our study was limited to individuals with AIS 1 or more injuries (the previous study included women with no injuries, an AIS of 0) and non-pregnant women were restricted to those aged 15 to 50 years (the previous study defined this group as aged 13 to 44 years). However, the above study and other previous studies have also reported that pregnant women receive minor injuries more often than non-pregnant women, which is similar to our results.

The reasons for fewer fatalities and lower injury severity among pregnant women compared with non-pregnant women in our study may be owing to the following. First, collisions were less severe among pregnant women than non-pregnant ones. Less frequent airbag deployment and pretensioner system activation in pregnant women may be due to lower velocity in a frontal collision, although there were no significant differences in DVTOTAL between the two groups. Furthermore, a significantly lower prevalence of sitting in the left front seat (driver’s position) suggested lower frequency of contact with the steering wheel among pregnant women. This tendency could partly be explained by adaptive changes in the daily activities of pregnant women, who tend to be cautious and refrain from potentially life-threatening behaviors. Second, pregnant women are more likely to seek medical attention after an MVC because of concern for the well-being of the fetus. Moreover, pregnant women tend to report an injury, in even a minor MVC, more often than non-pregnant women [10]. Third, the reason for the higher rate of hospital admission among pregnant women might not be owing to injury itself, but rather to the clinical need to conduct follow-up and monitor both the mother and fetus.

**Figure 1:**
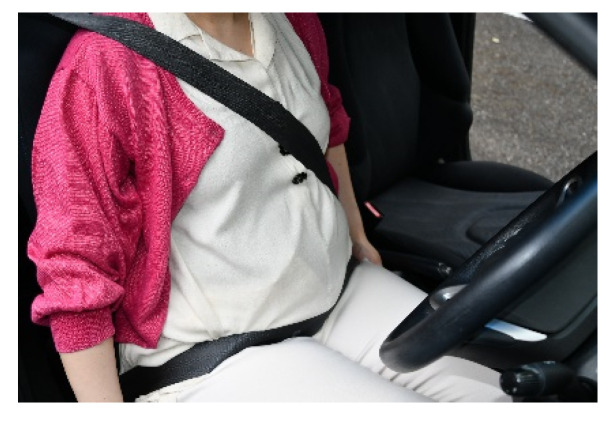
A pregnant woman at 30 weeks of gestation in the driver’s seat.

We found that the rate of AIS 2+ injuries of the abdomen was higher in pregnant women than in non-pregnant women, although the rate of sustaining AIS 2+ injuries in other body regions was lower in pregnant women owing to less severe collisions. In pregnant women, because the uterus extends to the central area of the abdomen, the abdomen is protruded according to the gestational age. When a pregnant woman at 30 weeks’ gestation sits in the driver’s seat, the distance between the steering wheel and the abdomen is approximately 10 cm shorter than for a non-pregnant woman (Figure 1). Thus, a pregnant woman is more likely to have contact between her abdomen and the steering wheel [13]. Furthermore, restrained pregnant passengers experience injury from external forces via the shoulder belt, even in minor to moderate frontal collisions [14]. Therefore, moderate and severe abdominal injuries are more common in pregnant women.

In non-pregnant women, seatbelt use was a negative influence and airbag deployment and higher collision velocity were positive influences on the likelihood of moderate and severe injuries. It is of great interest that these results concur well with the results of a similar previous study on pregnant women in which the OR of seatbelt use was 0.304, that of airbag deployment was 2.00, and that of DVTOTAL was 3.030 at 21–40 km/h, 13.469 at 41–60 km/h, and 44.564 at ≥61 km/h (Table 6) [5]. Seatbelt use is the most effective intervention available to reduce the likelihood of moderate and severe injuries in a collision. A meta-analysis of cohort studies suggested that the risk of any major injury was significantly lower among belted passengers as compared with unbelted passengers (relative risk 0.47) [15]. Therefore, improvement in seatbelt use among both pregnant and non-pregnant women is an effective intervention. As previously mentioned, not only seatbelt fastening but also its correct use should be promoted in safety education provided by health professionals [16]. In this study, the rate of seatbelt use among pregnant women did not differ significantly from that in non-pregnant women (70.0% vs. 70.4%). A previous report using the NASS-CDS database suggested that pregnant women had a significantly lower rate of seatbelt use than non-pregnant women (70.0% vs. 90.3%) [17]. The difference from our results might be owing to the differences in participants as ours were limited to those with AIS 1 or more. Seatbelts are a passive safety feature that reduces fatal or serious injuries in situations in which a collision is unavoidable. In addition to preventing the passenger from moving, the seatbelt system contains a pretensioner that tightens any slack in the belt webbing when a collision occurs and load limiters that minimize injuries caused by the belt. Because seatbelt use was an independent factor that prevented moderate and severe injuries, further development of the seatbelt system is warranted, such as ensuring appropriate load limiter settings for different seating positions, developing designs that fit all body sizes, and a warning system to remind passengers to use the seatbelt properly. Furthermore, a collision mitigation system that involves seatbelts would be useful as an active safety feature. The usefulness of electric seatbelt retractors, which pull in the webbing to restrain occupants just prior to a collision, has been demonstrated.

It is also suggested that speeding behavior was the main predisposing factor in collisions among hospitalized drivers. Recently, active safety features that prevent collisions have been developed. Automatic emergency braking gradually slows the vehicle down by sensing its position relative to other cars and road users. Widespread implementation of this type of system may help to reduce moderate and severe injuries in female passengers. However, awareness about the risks of speeding should be raised until automatic emergency braking systems are further developed [18]. Additionally, especially for non-pregnant women, some factors related to vehicle collisions influence the risk of moderate and severe injuries. Seat position (i.e., sitting in a rear seat), principal direction of force (i.e., force from the left lateral side), number of collisions, and rollovers positively influenced the likelihood of AIS 2+ injuries. As mentioned above, the collision severity was higher in non-pregnant women than in pregnant women. Therefore, collision characteristics can influence the mechanisms and outcomes of injury, especially in non-pregnant women. Healthcare professionals cannot affect the severity of the collision itself or intervene to improve factors related to the collision characteristics. Therefore, the authors consider that interventions to prevent moderate and severe injuries in non-pregnant women are more difficult to develop than interventions for pregnant women. Because collision-influencing factors for pregnant women are limited to seatbelt use, airbag deployment, and DVTOTAL, simple and effective interventions could be developed for this population. These factors could be improved using safety education provided by health professionals to pregnant women, which would improve the outcomes for the fetus in an MVC [18].

This study had several limitations. First, the NASS-CDS comprises reports of collisions in which one or more involved vehicles had been towed from the scene. Therefore, the database does not include reports of low-severity collisions. The objective of this study was to compare collision characteristics and injuries between pregnant women and non-pregnant women involved in an MVC. A sufficient number of non-pregnant women with moderate and severe injuries were included in the present sample. Therefore, we believe that this limitation is unimportant. Second, in this study, we used a US vehicle collision database. Because traffic situations and collision characteristics, such as passenger body size, vehicle size, and speed limits, vary worldwide, the present findings may not be generalizable to all countries. Additional research is required using data from international sources. Third, although we analyzed detailed collision factors in the present study, little passenger information (e.g., age, height, weight) was included. Therefore, the present findings may be insufficient to inform vehicle safety innovations. Additional studies are needed to analyze real-world collision cases and to investigate passenger health and kinematics immediately before collisions.

## 5. Conclusions

The study objectives were to conduct a detailed comparison of the characteristics and outcomes of MVCs involving pregnant and non-pregnant women, and to identify predictors of moderate-to-severe injuries in the two groups. We conducted a retrospective analysis using the NASS/CDS national database of collisions registered between 2001 and 2015. We found that collision severity, injury severity, and fatality rate were significantly lower in pregnant women than in non-pregnant women. This may partly be because pregnant women tend to exercise more caution in their daily activities. Because of abdominal protrusion, the rate of AIS 2+ injuries in the abdomen only was higher in pregnant women. For non-pregnant women, rear seat position, airbag deployment, multiple collisions, rollover, force from the left, and higher collision velocity positively predicted and seatbelt use and rear force negatively predicted AIS 2+ injuries.

This study extends previous research database studies [9,10,11,12] of collision and injury characteristics in pregnant and non-pregnant women by conducting a more in-depth comparison of such characteristics using detailed information from the NASS/CDS database. Only one previous study, by Manoogian (2015), has used the NASS/CDS database to compare pregnant and non-pregnant women [12]; however, Manoogian (2015) did not perform detailed comparisons of collision outcomes and MAIS scores, or examine predictors of injury in the two groups. Therefore, the present findings provide a more complete picture of the differences in collision characteristics and effects between pregnant and non-pregnant women, including new data on hospitalization and fatality outcomes. Although Manoogian (2015) compared injury rates for different body regions, we demonstrated for the first time that pregnant women had a higher rate of AIS 2+ abdominal injuries and a lower rate of AIS 2+ injuries in other body regions. Additionally, this is the first study to identify and compare predictors of injury in pregnant and non-pregnant women. As such, it represents an important contribution to the literature, because greater awareness of the factors that predict injury from MVCs will facilitate the development of interventions for pregnant and non-pregnant women.

The present findings have several potential applications. Our data could be used to inform interventions to improve seatbelt design and use in pregnant and non-pregnant women. However, any such measures must be tailored to the specific requirements of pregnant women. Our findings suggest that both vehicle innovations (e.g., active safety features) and education by healthcare professionals could reduce moderate and severe injuries in pregnant and non-pregnant women. In particular, interventions for pregnant women should focus on seatbelt use and reductions in collision velocity. Although better outcomes were observed for pregnant women than for non-pregnant women, the former showed a higher prevalence of AIS 2+ abdominal injuries. Therefore, interventions should focus on reducing abdominal injuries in this population.

The results of previous studies comparing pregnant and non-pregnant women on MVC characteristics and outcomes are inconsistent, and only two studies (the present study and [12]) have examined these characteristics in detail. Therefore, more studies are needed to confirm which factors affect moderate and severe MVC injuries in pregnant and non-pregnant women. More interventions by healthcare professionals are also needed to reduce MVCs in these two groups of women. There have been several recent innovations in the automotive industry (e.g., the development of autonomous driving). However, such increasingly complex systems must be rigorously tested. There is a need for the development of safety measures for occupants sitting in an upright position in a defined seat, and for passive and active safety measures that work in tandem. For example, our findings suggest that seatbelt use and collision velocity reduction are effective measures for preventing moderate and severe injuries in women; therefore, passive safety technologies for restraint and active safety features that slow down the vehicle and mitigate collisions are needed. Additionally, to prevent moderate and severe passenger injuries, interventions must be developed to encourage correct seatbelt use and reduce driving speed. It is likely that simple educational interventions provided by health professionals could help to reduce collisions in pregnant women.

## Figures and Tables

**Table 1:** Comparison of the background and collision characteristics between pregnant and non-pregnant women. DVTOTAL, delta-V total.

Item	Pregnant Women (*n* = 736)	Non-Pregnant Women (*n* = 21,874)	*p* Value
Age (yrs.)	28.1 ± 6.4	29.6 ± 10.3	<0.001
Height (cm)	164.1 ± 8.3	164.4 ± 8.3	0.533
Weight (kg)	74.6 ± 19.1	69.5 ± 18.6	<0.001
Seating position			<0.001
Front left	476 (64.7%)	15,245 (69.7%)	
Front right	206 (28.0%)	4766 (21.8%)	
Rear	52 (7.0%)	1839 (8.4%)	
Unknown	2 (0.3%)	24 (0.1%)	
Seatbelt use			0.160
Yes	515 (70.0%)	15,393 (70.4%)	
No	185 (25.1%)	5051 (23.1%)	
Unknown	36 (4.9%)	1430 (6.5%)	
Pretensioner			0.027
Acting	91 (12.4%)	3355 (15.3%)	
Not acting or Unknown	645 (87.6%)	18,519 (84.7%)	
Airbag deployment			<0.001
Yes	308 (41.8%)	9760 (44.6%)	
No	428 (58.2%)	9772 (44.7%)	
Unknown	0 (0%)	2342 (10.7%)	
Direction of forces			0.835
Front	334 (45.4%)	10,226 (46.8%)	
Right	82 (11.1%)	2327 (10.6%)	
Rear	50 (6.8%)	1379 (6.3%)	
Left	90 (12.2%)	2458 (11.2%)	
Unknown	180 (24.5%)	5484 (25.1%)	
DVTOTAL (km/h)			0.147
1–20	194 (26.3%)	5052 (23.1%)	
21–40	178 (24.2%)	6051 (27.7%)	
41–60	49 (6.7%)	1397 (6.4%)	
≥61	10 (1.4%)	355 (1.6%)	
Unknown	305 (41.4%)	9019 (41.2%)	
Crash times	1.6 ± 1.0	1.7 ± 1.0	0.152
Rollover			0.973
Yes	94 (12.8%)	2836 (13.0%)	
No	630 (85.6%)	18,700 (85.5%)	
Unknown	12 (1.6%)	338 (1.5%)	

**Table 2:** Comparison of outcomes and injury severity between pregnant and non-pregnant women. MAIS, maximum abbreviated injury severity; AIS, abbreviated injury scale.

Item	Pregnant Women (*n* = 736)	Non-Pregnant Women (*n* = 21,874)	*p* Value
Outcome			<0.001
Death	14 (1.9%)	822 (3.8%)	
Admission	264 (35.9%)	5133 (23.4%)	
Outpatient	458 (62.2%)	15,873 (72.6%)	
Unknown or diseased death	0 (0%)	66 (0.3%)	
MAIS	1 (1, 1)	1 (1, 2)	<0.001
Prevalence of AIS 2+ injuries			
Head	73 (9.9%)	2869 (13.1%)	0.011
Face	15 (2.0%)	627 (2.9%)	0.183
Neck	0 (0%)	58 (0.3%)	0.162
Chest	32 (4.3%)	1939 (8.9%)	<0.001
Abdomen	43 (5.8%)	1130 (5.2%)	0.416
Spine (neck)	14 (1.9%)	691 (3.2%)	0.054
Spine (chest)	6 (0.8%)	375 (1.7%)	0.062
Spine (abdomen)	7 (1.0%)	494 (2.3%)	0.018
Upper extremities	46 (6.3%)	2018 (9.2%)	0.006
Lower extremities	76 (10.3%)	2941 (13.4%)	0.014

**Table 3:** Comparison of the background and collision characteristics between non-pregnant women with MAIS 1 injuries and MAIS 2+ injuries. MAIS, maximum abbreviated injury severity; DVTOTAL, delta-V total.

Item	MAIS 1 (*n* = 15,073)	MAIS 2+ (*n* = 6798)	*p* Value
Age (yrs.)	29.6 ± 10.2	29.6 ± 10.3	0.706
Height (cm)	164.3 ± 8.2	164.6 ± 8.4	0.006
Weight (kg)	69.3 ± 18.2	70.0 ± 19.6	0.022
Seating position			<0.001
Front left	10,738 (71.2%)	4507 (66.3%)	
Front right	3225 (21.4%)	1541 (22.7%)	
Rear	1102 (7.3%)	737 (10.8%)	
Unknown	8 (0.1%)	16 (0.2%)	
Seatbelt use			<0.001
Yes	11,694 (77.6%)	3699 (54.4%)	
No	2511 (16.7%)	2540 (37.3%)	
Unknown	868 (5.8%)	562 (8.3%)	
Pretensioner			0.349
Acting	2335 (15.5%)	1020 (15.0%)	
Not acting or Unknown	12,738 (84.5%)	5781 (85.0%)	
Airbag deployment			<0.001
Yes	6484 (43.0%)	3276 (48.2%)	
No	7077 (47.0%)	2695 (39.6%)	
Unknown	1512 (10.0%)	830 (12.2%)	
Direction of forces			<0.001
Front	7026 (46.6%)	3199 (47.1%)	
Right	1609 (10.7%)	718 (10.6%)	
Rear	1162 (7.7%)	217 (3.2%)	
Left	1603 (10.6%)	855 (12.6%)	
Unknown	3673 (24.4%)	1811 (26.6%)	
DVTOTAL (km/h)			<0.001
1–20	4333 (28.7%)	718 (10.6%)	
21–40	4293 (28.5%)	1758 (25.8%)	
41–60	497 (3.3%)	900 (13.2%)	
≥61	57 (0.4%)	298 (4.4%)	
Unknown	3673 (24.4%)	1811 (26.6%)	
Crash times	1.6 ± 0.9	1.9 ± 1.2	<0.001
Rollover			<0.001
Yes	1608 (10.7%)	1228 (18.1%)	
No	13,322 (88.4%)	5378 (79.1%)	
Unknown	143 (0.9%)	195 (2.9%)	

**Table 4:** Comparison of outcomes between non-pregnant women with MAIS 1 injuries and MAIS 2+ injuries. MAIS, maximum abbreviated injury severity.

Item	MAIS 1 (*n* = 15,073)	MAIS 2+ (*n* = 6798)	*p* Value
Outcome			<0.001
Death	29 (0.2%)	793 (11.7%)	
Admission	916 (6.1%)	4197 (61.7%)	
Outpatient	14,087 (93.5%)	1783 (26.3%)	
Unknown or diseased death	41 (0.3%)	25 (0.4%)	

**Table 5:** Results of logistic regression analysis to predict moderate-to-severe injuries in non-pregnant women. DVTOTAL, delta-V total.

Variable	Odds Ratio	95% Confidence Interval	*p* Value
Height	1.004	0.997–1.010	0.248
Weight	1.005	1.002–1.007	0.001
Seating position			
Front left	Ref	Ref	
Front right	1.003	0.888–1.132	0.966
Rear	1.345	1.101–1.642	0.004
Seatbelt use	0.364	0.326–0.406	<0.001
Airbag deployment	1.297	1.160–1.451	<0.001
Direction of force			
Front	Ref	Ref	
Right	1.037	0.900–1.196	0.612
Rear	0.360	0.281–0.463	<0.001
Left	1.478	1.291–1.692	<0.001
DVTOTAL			
1–20	Ref	Ref	
21–40	2.504	2.235–2.805	<0.001
41–60	11.674	9.930–13.723	<0.001
≥61	43.220	29.902–62.472	<0.001
Crash time	1.277	1.206–1.351	<0.001
Roll over	1.759	1.420–2.178	<0.001

**Table 6:** Comparison of factors influencing moderate-to-severe injuries among pregnant women [5] and non-pregnant women. DVTOTAL, delta-V total.

Item	Odds Ratio	
	Pregnant Women Ref. [5]	Non-Pregnant Women
Seatbelt use	0.304	0.364
Airbag deployment	2.002	1.297
DVTOTAL		
1–20 (Ref)	-	-
21–40	3.030	2.504
41–60	13.469	11.674
≥61	44.564	43.220
Seating on rear seat (Ref: front left)	N/A	1.345
Direction force (Ref: front)		
Left	N/A	1.478
Rear	N/A	0.360
Crash time	N/A	1.277
Roll over	N/A	1.759

## Data Availability

The data presented in this study are available upon request from the corresponding author.
